# Available energy and nutrient apparent total tract digestibility of full-fat rice bran fed to gestating and lactating sows

**DOI:** 10.1093/jas/skag267

**Published:** 2026-08-26

**Authors:** Can Zhang, Bo Cheng, Lei Xue, Jianjun Zang

**Affiliations:** National Feed Engineering Technology Research Center, College of Animal Science and Technology, China Agricultural University, Beijing 100193, China; National Feed Engineering Technology Research Center, College of Animal Science and Technology, China Agricultural University, Beijing 100193, China; National Feed Engineering Technology Research Center, College of Animal Science and Technology, China Agricultural University, Beijing 100193, China; National Feed Engineering Technology Research Center, College of Animal Science and Technology, China Agricultural University, Beijing 100193, China

**Keywords:** available energy, full-fat rice bran, sow, substitution level

## Abstract

The substitution method was used to design four substitution levels (15%, 25%, 35%, 45%) in Exp. 1 and three substitution levels (15%, 25%, 35%) in Exp. 3, to determine the appropriate level of full-fat rice bran (FFRB) for replacing energy-supplying components in the basal diet for gestating sows (Exp. 1: gestation day, 35 ± 5 d; body weight, 194.6 ± 12.1 kg; parity, 3–4) and lactating sows (Exp. 3: body weight, 252.4 ± 13.6 kg; parity, 3–6). Based on optimal substitution levels identified in Exp. 1 and 3, test diets were formulated for Exp. 2 and 4, respectively. The available energy values of five FFRB sources (Exp. 2) and four FFRB sources (Exp. 4) were evaluated in gestating sows (gestation day, 35 ± 5 d; body weight, 197.9 ± 5.3 kg; parity, 3–4) and lactating sows (body weight, 270.0 ± 25.4 kg; parity, 3–6), respectively. Predictive equations for the available energy of FFRB were established for both reproductive stages. For gestating sows, the 35% substitution level group achieved the highest net energy (NE) value, with significantly greater energy retention as lipid (RE_L_) compared with other substitution groups (*P *< 0.05). In addition, the group exhibited the lowest coefficient of variation (CV) for digestible energy (DE), metabolizable energy (ME), and NE values. Therefore, a 35% substitution level of FFRB for energy components in the basal diet was optimal for gestating sows using the substitution method. For lactating sows, the 25% substitution level was optimal based on the lowest CV for DE and ME values. Lactating sows demonstrated greater nutrient digestibility than gestating sows; however, the energy utilization of FFRB was higher in gestating sows. In gestating sows, the DE values of five FFRB sources ranged from 15.22 to 15.90 MJ/kg dry matter (DM), ME values from 14.63 to 15.29 MJ/kg DM, and NE values were 10.77, 11.97, 12.58, 11.55, and 11.45 MJ/kg DM. In lactating sows, DE ranged from 13.28 to 13.97 MJ/kg DM, and ME from 11.13 to 12.19 MJ/kg DM. Fiber, ether extract (EE), and crude protein (CP) were predominant predictors of available energy of FFRB for sows. The predictive equations for DE, ME, and NE of FFRB in gestating sows were as follows: DE = 0.42 EE − 0.09 NDF + 11.20 (*R*² = 0.71, *P *< 0.01); ME = 0.70 DE − 0.02 Ash + 4.24 (*R*² = 0.79, *P *< 0.01); NE = 0.05 ST − 0.37 ADF + 14.75 (*R*² = 0.85, *P *< 0.01). The predictive equation for ME of FFRB in lactating sows was: ME = 1.12 DE + 0.06 CP − 0.03 NDF − 3.86 (*R*^2^ = 0.97, *P *< 0.01).

## Teaser

The optimal substitution level of full-fat rice bran (FFRB) for energy-supplying components in the basal diet was determined to be 35% for gestating and 25% for lactating sows using the substitution method. The predictive equation for NE of FFRB in gestating sows was as follows: NE = 0.05 ST − 0.37 ADF + 14.75 (*R*² = 0.85, *P *< 0.01).

## Introduction

Less expensive, alternative ingredients rich in fiber or in protein have been increasingly included in swine diets to reduce feed costs. Full-fat rice bran (FFRB), a by-product of the rice grain milling process (Li et al. 2018), is commonly used as an alternative energy ingredient in diets because it contains 15–22% oil (Zhang et al. 2022). Moreover, FFRB is abundant in vitamin E, vitamin B1, niacin, and various mineral elements (Saunders 1990). Extensive research on fiber nutrition has verified that properly regulated fiber concentrations and fiber sources benefit sow production, and FFRB can serve as a viable feed ingredient for sow diets (Guillemet et al. 2007; Quesnel et al. 2009). However, FFRB exhibits significant quality variability, as it is influenced by factors including rice variety, impurity content, moisture content, kernel plumpness, and processing precision (Lowell et al. 2015). To improve the utilization efficiency of FFRB and reduce feed costs, accurate evaluation of its nutritional value is essential. Currently, the in vivo method is internationally recognized as an effective approach for determining the available energy value of feed ingredients (Noblet et al. 2004). The substitution method is particularly suitable for determining the available energy of ingredients that cannot be directly fed to pigs. Due to antinutritional factors and palatability issues, FFRB cannot be used as the sole feed ingredient. Therefore, the substitution method is a preferred choice for determining the nutritional value of FFRB. The substitution level of the test ingredient is a critical factor influencing the accuracy of available energy determination by this method (Dong 2020). While higher substitution levels theoretically enhance precision, excessive inclusion may introduce significant nutritional disparities between basal and test diets; such compositional differences can compromise the accuracy of available energy assessment for the test ingredient (Villamide 1996). Currently, FFRB has been investigated in growing and finishing pigs; however, research on its application in sows remains limited. Available energy includes digestible energy (DE), metabolizable energy (ME), and net energy (NE), among which the NE evaluation system can represent the truly available energy in feed more accurately (Lyu 2021). However, determining NE by indirect calorimetry is difficult to implement in lactating sows. Therefore, this experiment investigated the appropriate level of FFRB replacement for energy-supplying components in the basal diet for gestating sows through respiratory metabolism trial, and for lactating sows through digestion-metabolism trial, using available energy value, coefficient of variation (CV), and nutrient digestibility as indicators. As the available energy value of ingredients is not fixed, formulating diets based on static data poses risks. An effective approach to address these issues is to establish a dynamic model that uses routine chemical composition to predict the available energy of feed ingredients (Noblet and Perez 1993). Therefore, based on the results of the substitution method for replacing energy-supplying components in the basal diet with FFRB, this article further determined the available energy values of FFRB from different sources and initially established available energy prediction equations for gestating and lactating sows.

## Materials and methods

All experimental protocols including animal care and use were approved by the Institutional Animal Care and Use Committee of China Agricultural University (AW50204202-1-2). All animal handling and experimental procedures complied with the FASS Guide for the Care and Use of Agricultural Animals in Research and Teaching. The experiments were conducted at the Fengning Swine Research Unit of China Agricultural University located in Chengde, Hebei, China.

### FFRB samples collection

Six FFRB samples (Table 1) from major rice-producing areas across China were collected in Experiment 1 (Exp. 1) and Experiment 2 (Exp. 2), including two from Heilongjiang Province (No.1 and No.5), one from Jilin Province (No.2), Guangdong Province (No.3), Zhejiang Province (No.4), and Liaoning Province (No.6), respectively.

**Table 1:** FFRB nutrient composition in Exp. 1 and Exp. 2 (%, DM basis).

Items	FFRB						
	No.1	No.2		No.3	No.4	No.5	No.6
**Origin**	**Heilongjiang**	**Jilin**		**Guangdong**	**Zhejiang**	**Heilongjiang**	**Liaoning**
**Nutrient compositions (measured values)**							
** GE, MJ/kg DM**	18.93	18.93	18.12		17.73	17.23	16.85
** DM**	89.53	90.00	91.35		91.35	90.31	90.74
** CP**	13.18	13.41	13.85		14.37	15.77	17.71
** Ash**	8.22	9.07	8.53		7.36	8.50	8.29
** NDF**	27.90	26.26	22.63		21.33	21.58	25.27
** ADF**	12.67	12.71	10.63		8.70	10.8	11.25
** OM**	81.31	80.93	82.83		84.33	81.81	82.45
** EE**	15.02	16.09	14.26		15.40	14.14	14.58
** Ca**	0.11	0.31	0.28		0.15	0.14	0.26
** P**	1.51	1.64	1.56		1.78	1.64	1.68
** ST**	27.25	14.55	25.9		26.11	21.98	20.66

Based on known proximate nutrients, five FFRB samples (Table 2) with varying ether extract (EE) contents were collected from feed processing or FFRB production manufacturers in major rice-producing areas of China in Experiment 3 (Exp. 3) and Experiment 4 (Exp. 4). These included one sample from Heilongjiang Province (No. 7), two from Jilin Province (Nos. 8 and 9), one from Jiangsu Province (No.10), and one from Beijing (No.11).

**Table 2:** FFRB nutrient compositions in Exp. 3 and Exp. 4 (%, DM basis).

Items	FFRB				
	No.7	No.8	No.9	No.10	No.11
Origin	Heilongjiang	Jilin	Jilin	Jiangsu	Beijing
**Nutrient compositions (measured values)**					
** GE, MJ/kg DM**	18.37	18.39	17.20	18.37	19.93
** DM**	89.00	88.65	89.71	89.77	90.93
** CP**	15.33	17.63	17.56	13.99	14.01
** Ash**	9.24	9.17	9.42	9.34	7.96
** ADF**	13.63	7.92	23.90	10.95	12.12
** NDF**	28.39	21.70	37.42	23.94	25.83
** OM**	79.26	79.47	80.29	80.43	82.97
** EE**	13.98	14.04	7.71	13.13	18.47

### Experimental animals and diets

Exp. 1: Respiratory calorimetry trials were conducted using open-circuit respiration chambers. Ten gestating sows (Landrace × Large White; gestation day = 35 ± 5 d) were selected based on initial body weight (194.6 ± 12.1 kg) and parity (3–4). Ten sows were divided into two groups of five sows each. A replicated 5 × 3 Youden square design was employed with five diets (basal diet and 15%, 25%, 35%, and 45% FFRB substitution level diets) and three successive periods. The experiment comprised six periods, each period lasted 10 d, consisting of 6-d dietary adaptation phase followed by a 4-d heat production (HP) measurement phase. During the first 5 d (d 1–5), gestating sows were housed in metabolism crates (1.7 × 0.7 × 1.4 m) at a temperature of 22 ± 2 °C. On the morning of d 6, sows were transferred to the chambers for the measurement of O_2_, CO_2_, and CH_4_ concentrations. On d 9, gestating sows were fasted; HP measured from 10:30 p.m. (d 9) to 6:30 a.m. (d 10) was designed as the fasting heat production (FHP). To allow a consistent time span for comparing FHP to total HP, the 8-h HP was extrapolated to a 24 h period (Zhang 2014). The gestating sows were fed equal-sized meals twice a daily at 8:30 a.m. and 3:30 p.m. Sows were fed at 628 kJ ME/kg BW^0.75^ d^−1^, which was approximately 1.5 times the maintenance energy requirement for gestating sows (Xue et al. 2025). Temperature was maintained at 20 ± 1 °C in the chambers and the relative humidity was controlled at 70 ± 5%.

Exp. 2: Based on the optimal substitution level of FFRB for gestating sows determined in Experiment 1 (35%), five FFRB samples from different sources were measured to establish prediction equations for the available energy of FFRB. Twelve gestating sows (Landrace × Large White; gestation day = 35 ± 5 d) were selected based on initial body weight (197.9 ± 5.3 kg) and parity (3–4). A replicated 6 × 3 Youden square design was employed with six diets and three successive periods. Each Youden square included six sows and six respiration chambers. The remaining respiratory calorimetry trial procedures were the same as those in Exp. 1.

Exp. 3: A digestibility and metabolism trial was conducted with 24 sows (Landrace × Large White) with parities ranging from 3 to 6 and an average body weight of 252.4 ± 13.6 kg. A randomized complete block design was adopted, sows were grouped into six blocks based on parity and initial body weight to form a randomized complete block design, which contained four treatments (basal diet and 15%, 25%, and 35% FFRB substitution level diets), with each block containing all four treatments and one sow per experimental unit (replicate). On d 107 of gestation, sows were transferred to farrowing crates and housed there until parturition. Each sow was offered three equal portions at 6:00 a.m., 10:00 a.m., and 3:30 p.m. Approximately 0.5 kg of feed was provided per sow on the day of parturition. Thereafter, the daily feed allowance was gradually increased by 1.0 kg per day until ad libitum feeding was attained. The pre-experimental period commenced on d 7 postpartum, during which basal diet and four FFRB test diets were fed. Total feces and urine samples were collected from d 14 to d 21 postpartum. Throughout the experiment, sows had free access to drinking water, and the ambient temperature was maintained between 20 °C and 23 °C.

Exp. 4: Based on the optimal substitution level of FFRB for lactating sows determined in Exp. 3 (25%), four FFRB samples from different sources were measured to establish prediction equations for the available energy of FFRB. Thirty sows (Landrace × Large White) were selected, with parities ranging from 3 to 6 and an initial body weight of 270.0 ± 25.4 kg. A randomized complete block design was adopted, sows were allocated into six blocks according to parity and initial body weight for a randomized complete block design, including five treatments (basal diet group and four FFRB test diet groups). Each block covered all five treatments, with one sow per replicate. All other digestibility and metabolism trial procedures were the same as those in Exp. 3.

### Experimental design and procedure

Exp. 1: The EE (15.02%) and Ash (8.22%) contents of FFRB No.1 were close to the average values of the six FFRB samples (Nos. 1–6). Therefore, FFRB No.1 was selected for Exp. 1 to ensure that the subsequent substitution experiment would reflect typical rather than extreme FFRB characteristics. Five diets were formulated: a corn-soybean meal basal diet and four test diets in which FFRB replaced energy-supplying components of the basal diet at substitution levels of 15%, 25%, 35%, and 45%. The diet compositions were shown in Table 3. Composition and nutritional content of all diets was adjusted according to the Nutrient Requirement of Swine in China (Li 2020).

**Table 3:** Diet compositions in Exp. 1 (%, as-fed basis).

Items	Substitution levels^1^				
	Basal diet	F15	F25	F35	F45
**Ingredients**					
** Corn**	85.73	72.87	64.30	55.72	47.15
** FFRB**	0.00	14.61	24.35	34.09	43.83
** Soybean meal**	11.67	9.92	8.75	7.59	6.42
** Dicalcium phosphate**	0.90	0.90	0.90	0.90	0.90
** Limestone**	0.60	0.60	0.60	0.60	0.60
** Salt**	0.60	0.60	0.60	0.60	0.60
** Premix^2^**	0.50	0.50	0.50	0.50	0.50
**Total**	100.00	100.00	100.00	100.00	100.00
**Analytical value**					
** GE, MJ/kg**	15.50	16.01	16.43	16.58	16.99
** CP**	12.42	12.95	12.59	13.19	13.18
** EE**	2.87	4.41	6.00	7.07	8.62
** NDF**	12.93	18.16	19.27	19.56	19.90
** ADF**	4.10	5.38	6.67	7.50	7.92
** Ash**	3.66	4.87	5.40	6.13	6.94
** OM**	83.66	82.88	83.05	82.76	82.45
** DM**	87.32	87.75	88.46	88.89	89.39

1 F15–F45 were test diets which replaced energy supplement in the basic diet with FFRB at levels of 15%, 25%, 35%, and 45%, respectively.

2 Premix provided per kilogram of complete feed: vitamin A, 11,000 IU; vitamin D_3_, 3,000 IU; vitamin E, 15.0 IU; vitamin K_3_, 1.6 mg; vitamin B_1_, 1.5 mg; vitamin B_2_, 3.0 mg; vitamin B_4_, 1.5 mg; vitamin B_12_, 0.02 mg; nicotinamide, 22.5 mg; pantothenic acid, 15.0 mg; folic acid, 2.5 mg; biotin, 0.2 mg; iron, 400.0 mg; copper, 16.5 mg; zinc, 75.0 mg; manganese, 35.0 mg; iodine, 1.0 mg; selenium, 0.3 mg.

Exp. 2: there were one basal diet and five test diets (Nos. 2–6 FFRB samples substituted 35% of the basal diet’s energy-supplying components) in this experiment. The nutrient components of the diets were shown in Table 4. The energy and nutrient levels of the test diets were adjusted according to the Nutrient Requirement of Swine in China (Li 2020).

**Table 4:** Diet compositions in Exp. 2 (%, as-fed basis).

Items	FFRB diets^1^					
	Basal diet	D2	D3	D4	D5	D6
**Ingredients**						
** Corn**	85.73	55.72	55.72	55.72	55.72	55.72
** FFRB**	0.00	34.09	34.09	34.09	34.09	34.09
** Soybean meal**	11.67	7.59	7.59	7.59	7.59	7.59
** Dicalcium phosphate**	0.90	0.90	0.90	0.90	0.90	0.90
** Limestone**	0.60	0.60	0.60	0.60	0.60	0.60
** Salt**	0.60	0.60	0.60	0.60	0.60	0.60
** Premix^2^**	0.50	0.50	0.50	0.50	0.50	0.50
**Total**	100.00	100.00	100.00	100.00	100.00	100.00
**Analytical value**						
** GE, MJ/kg**	15.51	16.80	16.50	16.58	16.52	16.50
** CP**	12.30	12.73	12.77	12.95	13.00	13.52
** EE**	2.42	6.88	7.34	6.79	6.56	6.55
** NDF**	9.87	14.08	12.31	9.83	11.07	14.04
** ADF**	3.54	6.71	5.57	4.71	5.47	5.84
** Ash**	3.83	6.03	5.65	5.33	5.71	5.69
** OM**	83.99	82.93	83.73	84.10	83.63	83.49
** DM**	87.82	88.96	89.38	89.43	89.34	89.18

1 D2, D3, D4, D5, and D6 were test diets containing 35% FFRB from five different sources.

2 Premix provided per kilogram of complete feed: vitamin A, 11,000 IU; vitamin D_3_, 3,000 IU; vitamin E, 15.0 IU; vitamin K_3_, 1.6 mg; vitamin B_1_, 1.5 mg; vitamin B_2_, 3.0 mg; vitamin B_4_, 1.5 mg; vitamin B_12_, 0.02 mg; nicotinamide, 22.5 mg; pantothenic acid, 15.0 mg; folic acid, 2.5 mg; biotin, 0.2 mg; iron, 400.0 mg; copper, 16.5 mg; zinc, 75.0 mg; manganese, 35.0 mg; iodine, 1.0 mg; selenium, 0.3 mg.

Exp. 3: Five FFRB samples (Nos. 7–11) were collected based on EE content. The EE (13.98%) and Ash contents (9.24%) of FFRB No.7 were close to both the average values of the five FFRB samples and the EE content of commonly used FFRB in current practice (Zhang and Lu 2020). Therefore, FFRB No.7 was selected as the test ingredient in Exp. 3 to allow the subsequent substitution effects to be attributed primarily to the inclusion rate rather than to feedstock variability. Three substitution levels (15%, 25%, and 35%) were designed in Exp. 3. Four diets were formulated, including one corn-soybean meal basal diet and three FFRB substitution diets (15%, 25%, and 35%). The composition of the diets was shown in Table 5, and both the energy and nutrient levels of the diets were adjusted according to the Nutrient Requirement of Swine in China (Li 2020).

**Table 5:** Diet compositions in Exp. 3 (%, fed basis).

Items	Substitution levels^1^			
	Basal diet	F15-2	F25-2	F35-2
**Ingredients**				
** Corn**	65.00	55.25	48.75	42.25
** Soybean meal**	32.00	27.20	24.00	20.80
** FFRB**	0.00	14.55	24.25	33.95
** Dicalcium phosphate**	0.70	0.70	0.70	0.70
** Salt**	0.80	0.80	0.80	0.80
** Limestone**	1.00	1.00	1.00	1.00
** Premix^2^**	0.50	0.50	0.50	0.50
**Total**	100.00	100.00	100.00	100.00
**Analytical value**				
** GE, MJ/kg**	16.27	16.45	16.58	16.63
** DM**	89.02	88.78	88.86	88.94
** CP**	19.57	18.55	17.57	16.42
** Ash**	4.99	6.18	6.67	7.40
** ADF**	4.83	6.25	6.92	7.02
** NDF**	13.96	15.38	15.69	16.64
** OM**	84.04	82.60	82.20	81.54

1 F15-2, F25-2, and F35-2 were test diets which replaced energy supplement in the basic diet with FFRB at levels of 15%, 25%, and 35%, respectively.

2 Premix provided per kilogram of complete feed: Vitamin A, 25,000 IU; Vitamin D_3_, 5,000 IU; Vitamin E, 12.5 I U; Vitamin K_3_, 1.0 mg; Thiamin, 8.0 mg; Pyridoxine 3.0 mg; Vitamin B_12_, 15 μg; Riboflavin, 6.0 mg; Niacin, 17.5 mg; D-Pantothenic acid, 12.5 mg; Folic acid, 0.25 mg; Biotin, 0.1 mg; Choline chloride, 0.4 mg; Fe, 165 mg; Cu, 16.0 mg; Zn, 30 mg; Mn, 30 mg; I, 0.3 mg; Se, 0.3 mg.

Exp. 4: five diets were formulated: one corn-soybean meal basal diet and four FFRB (Nos. 8–11) substitution diets (25%). The nutrient components of the diets were the same as the basal diet and the 25% FFRB substitution level diet in Exp. 3. The composition of the diets was shown in Table 6.

**Table 6:** Diet composition and nutrition levels in Exp. 4 (%, DM basis).

Items	Basal diet	FFRB diets			
		No.8	No.9	No.10	No.11
**Ingredients**					
** Corn**	65.00	48.75	48.75	48.75	48.75
** Soybean meal**	32.00	24.00	24.00	24.00	24.00
** FFRB**	0.00	24.25	24.25	24.25	24.25
** Dicalcium phosphate**	0.70	0.70	0.70	0.70	0.70
** Salt**	0.80	0.80	0.80	0.80	0.80
** Limestone**	1.00	1.00	1.00	1.00	1.00
** Premix^1^**	0.50	0.50	0.50	0.50	0.50
**Total**	100.00	100.00	100.00	100.00	100.00
**Analytical value**					
** GE, MJ/kg DM**	17.87	18.34	18.26	18.69	18.80
** CP**	21.70	21.11	19.46	20.97	17.12
** Ash**	6.52	6.51	7.45	6.82	5.81
** ADF**	4.37	5.87	9.70	6.60	6.25
** NDF**	11.43	15.11	19.53	16.34	17.37
** OM**	93.48	93.49	92.54	93.18	94.19
** EE**	3.57	4.23	2.99	3.84	5.25

1 Premix provided per kilogram of complete feed: Vitamin A, 25,000 IU; Vitamin D_3_, 5,000 IU; Vitamin E, 12.5 I U; Vitamin K_3_, 1.0 mg; Thiamin, 8.0 mg; Pyridoxine 3.0 mg; Vitamin B_12_, 15 μg; Riboflavin, 6.0 mg; Niacin, 17.5 mg; D-Pantothenic acid, 12.5 mg; Folic acid, 0.25 mg; Biotin, 0.1 mg; Choline chloride, 0.4 mg; Fe, 165 mg; Cu, 16.0 mg; Zn, 30 mg; Mn, 30 mg; I, 0.3 mg; Se, 0.3 mg.

### Sample preparation and chemical analyses

All FFRB and test diet samples (50 g) were collected from the upper, middle, and lower parts of each bag. These samples were mixed thoroughly and sub-sampled via the quartering method. The sub-samples were crushed and stored in sealed plastic bags at −4 °C until analysis.

In Exp. 1 and Exp. 2, during d 6 to d 10, all feces from each gestating sow were collected daily at 8:30 and stored at −20 °C. Urine was also collected daily at 8:30 for each gestating sow using plastic buckets containing 50 mL of 6 mol/L HCl. The collected urine was filtered through cotton gauze, and 10% of the daily urine volume was stored at −20 °C. Urine was collected separately during the fasting period (d 10) for the calculation of FHP.

**Figure 1:**
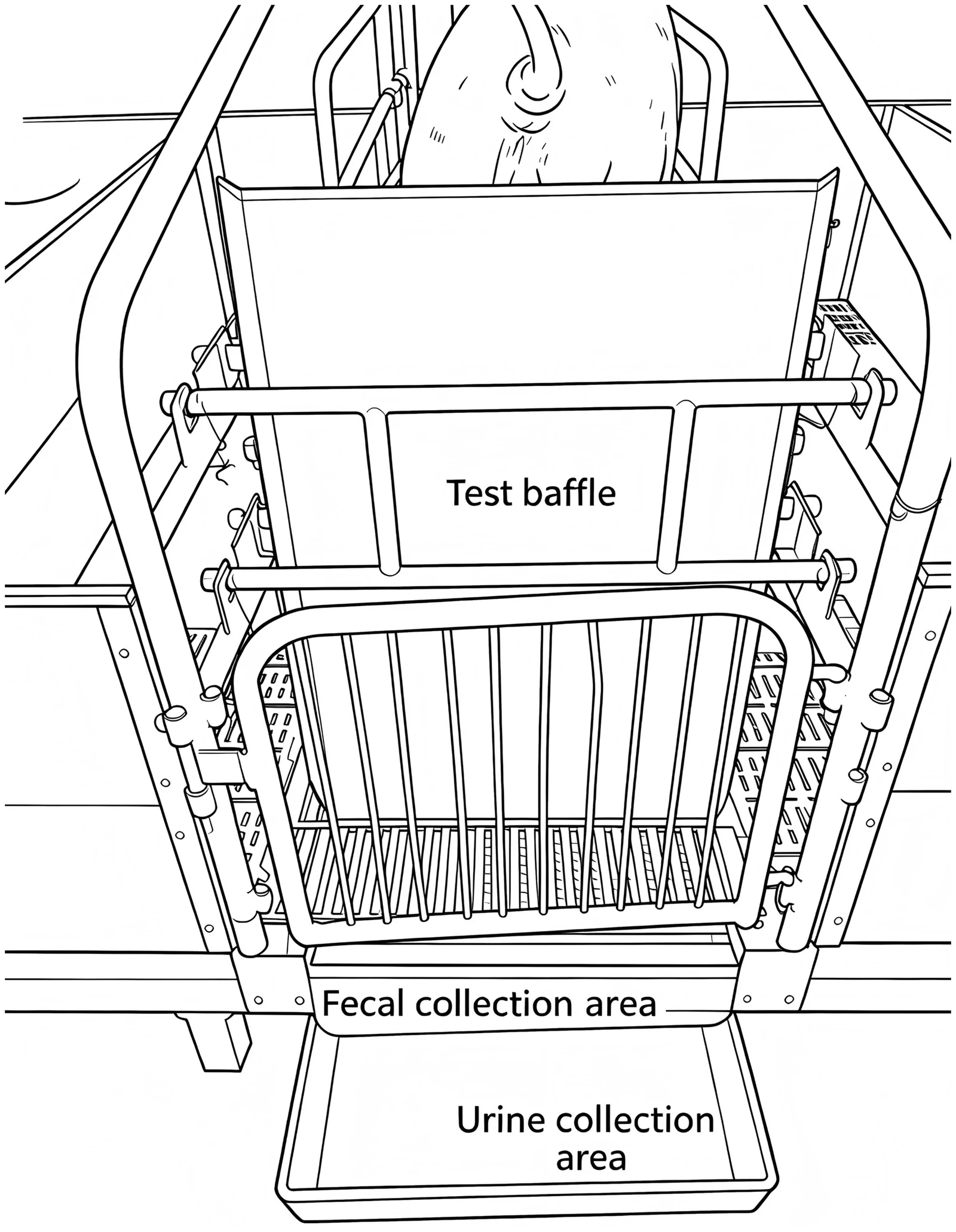
The feces and urine collection devices for lactating sows in Exp. 3 and Exp. 4.

In Exp. 3 and Exp. 4, feces from lactating sows were collected immediately after defecation and placed into plastic bags. All urine samples in the urine collection basins were collected every afternoon after feeding from d 14 to d 21 post-partum. The processing procedures for the remaining urine samples were the same as described above. The feces and urine collection devices (Patent No. ZL202322939161.4) for lactating sows were based on a conventional semi-slatted floor farrowing crate with confinement stalls (Figure 1). In addition to the crate, the system mainly consisted of a urine deflection device and a feces and urine collection device. The urine deflection device was positioned above the slatted floor to separate the connecting corridor from the sow’s excretion area. The urine deflection plate was equipped with side panels; one side of each side panel was connected to the urine deflection plate, while the other side extended toward the sow’s excretion area and abutted the farrowing crate. The feces and urine collection device were placed beneath the slatted floor. This collection device comprised a urine collection tray and a filter tray. The urine collection tray was situated on the bottom surface of the sow’s excretion area and featured a collection chamber that communicated with the sow’s excretion area. The filter tray was placed on top of the urine collection tray to collect feces.

At the end of each period in Exp. 1–4, daily fecal samples from each respective experiment were thawed and mixed; a sub-sample (300 g) from each respective experiment was then stored for subsequent analysis. In Exp. 1 and 2, daily urine samples from each respective experiment were thawed and mixed, and two sub-samples (50 mL each) were stored for subsequent analysis. Fecal sub-samples were oven-dried at 65 °C for 72 h and then ground to pass through a 40-mesh sieve.

Samples of FFRB, diets and feces were analyzed for DM (Method 934.01), Ash (Method 942.05) and EE (method 920.39) according to the procedures of the Association of Official Analytical Chemists (AOAC International 2016). Total nitrogen (N) was determined by method 976.05 (AOAC International 2016), and CP was calculated as N × 6.25. The contents of NDF and ADF was determined in accordance with Van Soest et al. (1991). Starch content was determined according to Method 996.11 (AOAC International 2016). Calcium (Ca) and phosphorus (P) concentrations were determined based on Chinese national standards GB/T 6436-2002 and GB/T 6437-2002, respectively (General Administration of Quality Supervision and Inspection and Quarantine of the People’s Republic of China 2002). The GE of FFRB, diets, feces, and urine samples was determined using an isoperibol calorimeter (Parr 6400 Calorimeter, Moline, IL, USA). The organic matter (OM) concentration was calculated as DM—Ash. The Amino acids (AA) contents of FFRB samples and diets were analyzed using an AA analyzer (Hitachi L-8900; Hitachi Ltd., Tokyo, Japan) and high-performance liquid chromatography (Agilent 1200 Series; Agilent Technologies Inc., Santa Clara, CA, USA) according to GB/T 18246-2000 (General Administration of Quality Supervision and Inspection and Quarantine of the People’s Republic of China 2019).

### Calculations

1. Total heat production (THP) and FHP of pigs were calculated for each day by gas exchange volumes and urinary N loss according to the following equation (Brouwer 1965):
  $$\mathrm{THP}\;\mathrm{or}\;\mathrm{FHP}\;(\mathrm{kJ})=16.1753\;\times O_{2}\;(L)+5.0208\times {\mathrm{CO}}_{2}\;(L)-2.1673\times {\mathrm{CH}}_{4}\;(L)-5.9873\times \mathrm{urinary}\;N\;(g)$$
2. The respiratory quotient (RQ) was calculated according to the following equation (Noblet et al. 1994):
  $$\mathrm{RQ}={\mathrm{CO}}_{2}(L/d)/O_{2}(L/d)$$
3. Retained energy (RE), retained energy as protein (RE_P_), and retained energy as lipid (RE_L_) in various diets were calculated according to the following equations (Noblet et al. 1994):
  RE (kJ)=ME intake (MJ)-THP (MJ)
   
  $${\mathrm{RE}}_{P}\;(\mathrm{kJ})=(\mathrm{nitrogen}\;\mathrm{intake},\;g-\mathrm{fecal}\;\mathrm{nitrogen},\;g-\mathrm{urine}\;\mathrm{nitrogen},\;g)\times 6.25\times 23.86\;(\mathrm{kJ}/g)$$
   
  $${\mathrm{RE}}_{L}\;(\mathrm{kJ})=\mathrm{net}\;\mathrm{deposition}\;\mathrm{energy}\;(\mathrm{kJ})-\mathrm{protein}\;\mathrm{deposition}\;\mathrm{energy}\;(\mathrm{kJ})$$
4. The apparent total tract digestibility (ATTD) of diets and FFRB in Exp. 1 and Exp. 2 was calculated according to the equations provided by Adeola et al. (2001):
  ATTD of dietary nutrients (%)=(total amount of nutrient intake-total amount of nutrient in feces)/total amount of nutrient intake
   
  ATTD of FFRB nutrients (%)=(ATTD of a certain nutrient in the test diet-(100%-X%)×ATTD of a certain nutrient in the basal diet
  Where X% represented the percentage of FFRB in the test diet.
5. The ATTD of diets and FFRB in Exp. 3 and Exp. 4 was calculated according to the following equation (Dong 1996):
  ATTD of FFRB = (content of nutrient in the test diet) × ATTD of nutrient in the test diet) − (1 − X%) × (nutrient content in basal diet × ATTD of nutrient in the basal diet)/(X% × content of nutrient in FFRB) × 100
  where X% was the percentage of FFRB to be tested in the test diet.
6. The DE and ME (Adeola 2001), and NE (Noblet et al. 1994) concentration of diets were calculated according to the following equations:
  DE (MJ/kg, DM)=(GE intake, MJ-fecal energy, MJ)/DM intake, kg
   
  ME (MJ/kg, DM)=(GE intake, MJ-fecal energy, MJ-urine energy, MJ-CH4 energy, MJ)/DM intake, kg
   
  NE (MJ/kg, DM)=(ME intake, MJ-THP, MJ+FHP, MJ)/DM, kg
7. The DE, ME and NE of FFRB were calculated according to the following equations (Xue et al. 2025):
  DE (MJ/kg, DM)=[DE of the test diet-DE of the basal diet×(1-X1%)]/X1%/X2%
   
  ME (MJ/kg, DM)=[ME of the test diet-ME of the basal diet×(1-X1%)]/X1%/X2%
   
  NE (MJ/kg, DM)=[NE of the test diet-NE of the basal diet)×(1-X1%)]/X1%/X2%

In these equations, X1% was the level of FFRB replacing the energy components of the basal diet; X2% was the level of energy components (corn and soybean meal) in the basal diet.

### Statistical analyses

All data were subjected to analysis of variance (ANOVA) using SAS software (version 9.4, SAS Institute Inc., Cary, NC, USA) via the MIXED procedure. Dietary treatment was treated as a fixed factor in the statistical mixed model. For Exp. 1 and Exp. 2, individual pig and experimental period were included as random effects; for Exp. 3 and Exp. 4, block was specified as the random effect, and each sow was defined as the experimental unit. The FFRB replacement level dependent effects (linear and quadratic) were analyzed using multivariate analysis in Exp.1 and Exp.3. Differences were considered statistically significant at *P *< 0.05, and tendencies were identified when 0.05 ≤ *P *< 0.10.

Correlation analysis was conducted using the PROC CORR procedure in SAS software to investigate the correlations between DE, ME, NE, and nutrient contents of FFRB. Meanwhile, stepwise regression analysis was performed via the PROC REG procedure in SAS software to screen the optimal fitting parameters and establish prediction equations for the available energy values of FFRB. The multiple coefficient of determination (*R*²), root mean square error (RMSE), and *P* value were used as evaluation criteria for the optimal prediction equations.

## Results

### ATTD of nutrients and energy balance of diets in Exp. 1

As the substitution level of FFRB increased, the ATTD of dietary DM, CP, ADF, and OM decreased linearly and quadratically (*P *< 0.01), the ATTD of dietary NDF decreased linearly (*P *< 0.01), while the ATTD of dietary EE increased linearly and quadratically (*P *< 0.01) (Table 7). Fecal output increased linearly and quadratically with the increase in substitution level (*P *< 0.01). As the substitution level of FFRB increased, the ME intake decreased linearly (*P *< 0.01) (Table 8). Except for the basal diet group, the RE_L_ of gestating sows in all test groups was negative, and it was significant higher in the 35% group (*P *< 0.05); meanwhile, RE in the 35% group was highest compared with the other test diets (*P *< 0.05). As the substitution level of FFRB increased, the dietary ME decreased linearly and quadratically (*P *< 0.01), the dietary NE first increased and then decreased with the increasing substitution level (*P *< 0.5), peaking at the 35% FFRB substitution level. Dietary treatments had a significant effect on the CH₄E/DE and ME/DE (*P *< 0.01), and a trend was observed for UE/DE (*P *< 0.1).

**Table 7:** Effects of different substitution levels on ATTD and nitrogen balance of diets fed to gestating sows in Exp. 1.

Items	Basal diet	Substitution level diets				SEM	Treatment	*P-*value	
		15%	25%	35%	45%			Linear	Quadratic
**DMI, kg/d**	2.19	2.20	2.27	2.23	2.21	0.33	0.97	0.75	0.83
**ATTD, %**									
** DM**	90.5	88.0	84.8	84.6	82.2	0.64	<0.01	<0.01	<0.01
** CP**	87.7	87.3	84.7	84.1	82.3	0.52	<0.01	<0.01	<0.01
** NDF**	62.7	61.8	60.7	56.6	51.90	1.71	0.21	0.02	0.05
** ADF**	72.0	60.2	57.7	55.8	52.0	1.75	<0.01	<0.01	<0.01
** OM**	92.6	90.5	87.9	87.5	85.5	0.53	<0.01	<0.01	<0.01
** EE**	59.7	69.0	74.2	80.2	82.2	1.72	<0.01	<0.01	<0.01
**Nitrogen balance, g/d**									
** Intake**	49.81	52.04	51.63	52.93	52.21	0.79	0.81	0.32	0.50
** Feces output**	6.07	7.79	10.03	10.09	11.69	0.42	<0.01	<0.01	<0.01
** Urine output**	19.26	20.79	21.59	20.68	21.96	0.86	0.90	0.40	0.67
** Retention**	24.48	23.45	20.01	22.16	18.57	1.28	0.60	0.15	0.36

DMI, dry matter intake.

**Table 8:** Effects of different substitution levels on energy balance of diets fed to gestating sows in Exp. 1.

Items	Basal diet	Substitution level diets				SEM	Treatment	*P-*value	
		15%	25%	35%	45%			Linear	Quadratic
**Energy balance, kJ/kg BW^0.75^/d**									
** ME intake**	629.14	612.98	588.49	579.10	568.78	9.61	0.26	0.02	0.07
** THP**	539.14	519.91	520.80	518.44	525.64	6.69	0.88	0.56	0.58
** FHP**	377.10	378.30	401.09	383.97	382.81	6.38	0.79	0.71	0.67
** RE_P_**	70.90	62.11	55.36	67.22	53.74	3.50	0.49	0.11	0.28
** RE_L_**	19.10	−13.25	−12.33	−1.44	−10.60	30.32	0.03	0.24	0.48
** RE**	90.00	48.86	43.03	65.78	43.14	11.77	0.01	0.13	0.32
**RQ**									
** Fed state**	1.00	0.96	0.95	0.97	0.94	0.01	0.73	0.24	0.48
** Fasted state**	0.80	0.79	0.77	0.81	0.78	0.01	0.90	0.80	0.94
**Available energy values, MJ/kg DM**									
** DE**	16.17	16.09	16.05	16.00	15.97	0.05	0.52	0.55	0.77
** ME**	14.78	14.49	13.99	13.82	13.34	0.12	0.35	<0.01	<0.01
** NE**	9.57	9.31	9.74	9.78	8.79	0.15	0.20	0.05	0.02
**Energy utilization, %**									
** UE/DE**	7.23	9.87	11.18	10.41	12.27	1.52	0.07	0.95	0.91
** CH_4_E/DE**	0.67	0.52	0.48	0.37	0.46	0.03	<0.01	<0.01	<0.01
** ME/DE**	92.1	89.6	88.3	89.2	87.30	0.82	<0.01	<0.01	<0.01
** NE/ME**	64.75	64.25	69.62	70.77	65.89	2.95	0.38	0.36	0.04

UE, urine energy; CH_4_E, CH_4_ energy.

### ATTD of nutrients and energy balance of FFRB in Exp. 1

Numerically, the 35% substitution level group had the highest available energy values and the lowest CV for available energy values of FFRB (Table 9). Specifically, compared with 15%, 25%, and 45% substitution level groups, the available energy CV of FFRB in the 35% substitution level group was reduced by 4.3%, 3.8%, and 2.2% (DE); 4.1%, 4.0%, and 1.8% (ME); 6.8%, 5.6%, and 3.9% (NE), respectively.

**Table 9:** ATTD and energy values of FFRB fed to gestating sows in Exp. 1.

Items	Substitution level diets				SEM	Treatment	*P-*value	
	15%	25%	35%	45%			Linear	Quadratic
**ATTD, %**								
** DM**	73.5	67.5	73.8	72.0	1.67	0.59	0.99	0.99
** OM**	78.0	73.7	78.2	76.8	1.42	0.67	1.00	0.84
** NDF**	57.3	54.8	45.4	38.6	6.38	0.75	0.27	0.54
** ADF**	26.9	24.7	27.5	25.8	6.67	0.24	0.05	0.12
** CP**	80.3	75.9	85.0	75.8	1.86	0.28	0.19	0.35
** EE**	34.9	37.8	38.2	39.6	3.92	0.74	0.30	0.57
**Available energy values, MJ/kg DM**								
** DE**	15.47	16.04	16.50	16.31	0.19	0.30	0.12	0.16
** ME**	14.46	14.59	15.51	15.05	0.41	0.35	0.71	0.71
** NE**	10.56	10.23	11.38	10.58	0.62	0.74	0.76	0.76
**Energy utilization, %**								
** ME/DE**	93.47	90.96	94.00	92.27	2.28	0.20	0.19	0.24
** NE/ME**	73.03	70.12	73.37	70.30	3.23	0.85	0.50	0.66
**CV, %**								
** DE**	6.8	6.3	2.5	4.7				
** ME**	6.2	6.1	2.1	3.9				
** NE**	11.4	10.2	4.6	8.5				

### ATTD of nutrients and energy balance of diets and FFRB in Exp. 2

The dietary ATTD of NDF and ADF, as well as nitrogen intake of gestating sows, were significantly affected by different FFRB supplemental diets (*P *< 0.01), but no other differences were observed among test diets (Table S1). The ME intake of gestating sows in the basal diet group (690.50 kJ/kg BW^0.75^/d) was significantly higher than that in all FFRB diet groups (*P *< 0.01) (Table S2). No significant differences were detected in DE, ME, and NE values or energy utilization among the different diets.

There were significant differences in the ATTD of NDF, ADF, and CP among different FFRB samples (*P *< 0.01) (Table 10). The ATTD of ADF in No. 4 FFRB was significantly higher than that in the other four samples (*P *< 0.01), whereas the ATTD of CP in No.6 FFRB was significantly lower than that in the others (*P *< 0.01). The values of DE, ME, and NE of five FFRB samples were 15.22–15.90 MJ/kg DM, 14.63–15.29 MJ/kg DM, and 10.77–12.58 MJ/kg DM, respectively. No significant differences were found in energy values or energy utilization among the five FFRB samples.

**Table 10:** ATTD of nutrients, available energy value, and energy utilization of FFRB fed to gestating sows in Exp. 2.

Items	FFRB diets					SEM	*P*-value
	No.2	No.3	No.4	No.5	No.6		
**ATTD, %**							
** DM**	80.7	82.9	84	83.1	82.4	0.38	0.12
** OM**	87.2	88.6	88.8	88.9	88.7	0.34	0.42
** NDF**	23.7^abc^	20.9^bc^	26.8^ab^	20.0^c^	28.6^a^	1.12	<0.01
** ADF**	22.9^b^	21.5^b^	35.9^a^	21.2^b^	22.2^b^	1.16	<0.01
** CP**	77.1^a^	77.6^a^	77.0^a^	69.5^b^	64.4^c^	1.12	<0.01
** EE**	35.6	37.5	36.4	35.9	34.5	0.34	0.09
**Available energy values, MJ/kg DM**							
** DE**	15.76	15.29	15.90	15.34	15.22	0.19	0.30
** ME**	14.88	14.74	15.29	14.89	14.63	0.24	0.28
** NE**	10.77	11.97	12.58	11.55	11.45	0.57	0.12
**Energy utilization, %**							
** ME/DE**	94.94	96.4	96.16	97.07	96.12	2.28	0.20
** NE/ME**	72.38	81.21	82.28	77.57	78.26	3.23	0.35

a,b,c Different lowercase letters within the same row indicate significant differences (*P *< 0.05), while the same or no letters indicate no significant differences (*P *> 0.05).

### Correlation between available energy value and chemical composition of FFRB in Exp. 2

The GE of FFRB was significantly negatively correlated with its CP (*r* = −0.91, *P *< 0.05), ST was significantly negatively correlated with NDF (*r* = −0.92, *P *< 0.05), and ADF was positively correlated with Ash (*r* = 0.93, *P *< 0.05) (Table S4). In addition, the NE value of FFRB was significantly negatively correlated with ADF (*r* = −0.92, *P *< 0.05), whereas the DE value of FFRB was significantly positively correlated with EE (*r* = 0.94, *P *< 0.05). The predictive equations for DE, ME, and NE of FFRB were established under the conditions of this research (Table 11): DE = 0.42 EE − 0.09 NDF + 11.20 (*R*² = 0.71, *P *< 0.01); ME = 0.70 DE − 0.02 Ash + 4.24 (*R*² = 0.79, *P *< 0.01); NE = 0.05 ST − 0.37 ADF + 14.75 (*R*² = 0.85, *P *< 0.01).

**Table 11:** Stepwise regression equation of available energy of FFRB fed to gestating sows in Exp. 2.

No.	Equation for FFRB (*n* = 5)	RMSE	*P-*value	*R* ^2^
**1**	DE = 0.42 EE − 0.09 NDF + 11.20	0.27	<0.01	0.71
**2**	ME = 0.70 DE − 0.02 Ash + 4.24	0.18	<0.01	0.79
**3**	NE = 0.05 ST − 0.37 ADF + 14.75	0.13	<0.01	0.85

### ATTD of nutrients and available energy of diets in Exp. 3

As the substitution level of FFRB increased, the dietary ATTD of CP, DM, OM, NDF decreased linearly and quadratically (*P *< 0.01) (Table 12), the dietary ATTD of ADF first increased and then decreased (*P *< 0.01), peaking at the 25% FFRB substitution level. Dietary DE and ME decreased linearly and quadratically as the substitution level increased (*P *< 0.05). No significant difference was found in the energy utilization among all treatment groups.

**Table 12:** Effects of various treatments on nutrient ATTD and available energy value of diets fed to lactating sows in Exp. 3 (%, DM basis).

Items	Basal diet	Substitution level diets			SEM	Treatment	*P*-value	
		15%	25%	35%			Linear	Quadratic
**DMI, kg/d**	7.63	7.03	7.22	7.00	0.58	0.18	0.13	0.25
**ATTD, %**								
** CP**	95.5	94.3	93.3	91.7	0.41	<0.05	<0.01	<0.01
** DM**	95.6	93.9	92.4	90.4	0.32	<0.05	<0.01	<0.01
** OM**	96.4	94.9	93.7	92.0	0.39	<0.05	<0.01	<0.01
** NDF**	88.4	76.6	79.9	72.6	0.74	<0.05	<0.01	<0.01
** ADF**	88.9	60.4	78.8	70.6	1.02	<0.05	0.12	<0.01
**Available energy values, MJ/kg DM**								
** DE**	16.12	16.10	15.87	15.84	0.35	<0.05	<0.01	<0.01
** ME**	15.55	15.44	15.08	14.86	0.58	<0.05	<0.01	0.04
**Energy utilization, %**								
** ME/DE**	96.46	95.93	95.02	95.14	0.12	0.85	0.35	0.28

DMI, dry matter intake

### ATTD of nutrients and available energy of FFRB in Exp. 3

The ATTD of ADF in FFRB first increased and then decreased as the substitution level increased (*P *< 0.01), peaking at the 25% substitution level. Except for ADF, the ATTD of other nutrients and available energy of FFRB peaked at the 25% substitution group among three substitution level groups (Table 13). No significant difference was found in the ME/DE of FFRB among all groups. The 25% substitution level group had the lowest CV for DE and ME of FFRB.

**Table 13:** Effects of different substitution levels on nutrient ATTD and available energy value of FFRB fed to lactating sows in Exp. 3.

Items	Substitution level diets			SEM	Treatment	*P-*value	
	15%	25%	35%			Linear	Quadratic
**ATTD, %**							
** CP**	86.2	86.5	83.5	1.33	0.76	0.16	0.36
** DM**	81.9	82.3	79.8	1.12	0.7	0.11	0.27
** OM**	84.4	85.4	84.2	0.48	0.86	0.12	0.29
** NDF**	49.6	53.2	49.9	1.86	0.19	0.23	0.23
** ADF**	43.8	45.2	42.5	1.37	0.25	<0.01	<0.01
**Available energy values, MJ/kg DM**							
** DE**	15.44	15.59	15.35	0.25	0.75	0.44	0.51
** ME**	13.95	14.20	13.92	0.30	0.67	0.58	0.42
**Energy utilization, %**							
** ME/DE**	90.35	91.08	90.68	0.76	0.75	0.75	0.28
**CV, %**							
** DE**	10.69	4.43	5.28				
** ME**	14.57	5.02	5.39				

### ATTD of nutrients, available energy, and energy utilization of diets and FFRB in Exp. 4

There were significant differences in ATTD of dietary ADF, DE, and ME among different FFRB diets groups (*P *< 0.05) (Table S3). There were no significant differences in the ATTD of nutrients or energy values among the four types of FFRB samples (Table 14). Numerically, No.11 FFRB had the highest ATTD of OM and DM. DE and ME of four FFRB samples were 13.28–13.97 MJ/kg DM and 11.13–12.19 MJ/kg DM. Among them, No.8 FFRB exhibited the highest DE (13.97 MJ/kg DM), ME (12.19 MJ/kg DM), and ME/DE value (87.26%).

**Table 14:** ATTD of nutrients, available energy value, and energy utilization of FFRB fed to lactating sows in Exp. 4 (%, DM basis).

Items	FFRB diets				SEM	*P*-value
	No.8	No.9	No.10	No.11		
**ATTD, %**						
** CP**	85.5	85.0	85.9	84.5	1.61	0.76
** DM**	79.0	79.6	79.4	80.7	0.82	0.66
** OM**	84.8	83.3	83.6	84.9	1.54	0.48
** NDF**	47.8	46.9	49.8	45.8	1.83	0.32
** ADF**	43.8	44.4	45.7	42.4	2.19	0.25
**Available energy values, MJ/kg DM**						
** DE**	13.97	13.78	13.28	13.84	0.51	0.63
** ME**	12.27	11.37	11.30	12.29	1.93	0.81
**Energy utilization, %**						
** ME/DE**	87.83	82.51	85.09	88.80	1.42	0.25

### Correlation between available energy value and chemical composition of FFRB in Exp. 4

A significant positive correlation was observed between DE and ME of FFRB (*r* = 0.99, *P *< 0.05) (Table S5). GE of FFRB was significantly positively correlated with EE (*r* = 0.98, *P *< 0.05), whereas a significant negative correlation existed between GE and Ash (*r* = −0.91, *P *< 0.05). In addition, a significant positive correlation was found between NDF and ADF of FFRB (*r* = 0.99, *P *< 0.05); both NDF and ADF of FFRB were negatively correlated with other nutrient components. Moreover, EE of FFRB was significantly positively correlated with DE, ME, and GE, but negatively correlated with other nutrient components.

The ME prediction equation for FFRB in lactating sows was established based on the present results (Table 15): ME = 1.12 DE + 0.06 CP − 0.03 NDF − 3.86 (*R*² = 0.97, *P *< 0.01).

**Table 15:** Stepwise regression equation of available energy of FFRB fed to lactating sows in Exp. 4.

Equation for FFRB (*n* = 4)	RMSE	*P*-value	*R* ^2^
**ME = 1.12 DE + 0.06 CP – 0.03 NDF – 3.86**	0.18	<0.01	0.97

## Discussion

### Effects of substitution levels on ATTD of nutrients in diets and FFRB in gestating and lactating sows

Except for EE, the ATTD of dietary nutrients in gestating sows decreased with increasing substitution levels, which may be attributed to the elevated dietary fiber content caused by the increased FFRB level (Kass et al. 1980). The high fiber content likely accelerated the passage rate of chyme through the intestine, thereby hindering contact between digestive enzymes and chyme and ultimately reducing nutrient ATTD (Son et al. 2023). The ATTD of EE of diets increased linearly and quadratically, which was also reported in a previous study (Zhang et al. 2025). Elevated EE intake can stimulate the secretion of bile acids and trypsin, which combine with fat in the digestive tract to form chylomicrons, effectively promoting lipid digestion and absorption (Schoeler and Caesar 2019). The average ATTD of NDF of FFRB in gestating sows (49.03%) was higher than that reported by Li et al. (2018) for growing pigs (32.6%). This discrepancy in digestibility may be attributed to the different digestive physiological characteristics of pigs at various physiological stages. Gestating sows have a more well-developed intestinal tract than growing pigs, resulting in improved fiber digestive capacity (Jørgensen et al. 2007). In addition, fecal nitrogen excretion increased linearly and quadratically with the increasing substitution ratio, which may be due to the elevated dietary fiber content promoting the conversion of urinary nitrogen to fecal nitrogen (Chen et al. 2023).

In a previous study by Casas and Stein (2017), gestating sow diets contained 40% FFRB, and the reported ATTD values of dietary DM (82.62%), OM (87.09%), NDF (49.22%), and ADF of FFRB (30.49%) were lower than those observed in the 35% FFRB substitution level group of our Exp. 1. Meanwhile, the ATTD of DM, OM, CP, NDF, and ADF of FFRB in the 35% substitution group was higher than that in the 45% substitution group. These results indicate that the high EE content in the 35% substitution group might counteract the adverse effects of high fiber content on FFRB nutrient digestibility. However, gestating sows have a certain tolerance to dietary fiber. Consistent with the finding in Exp. 1, as FFRB substitution levels increased to 40% and 45%, dietary fiber content exceeded the tolerance range of gestating sows, thereby reducing ATTD of CP and EE (Degen et al. 2007; Wilfart et al. 2007).

The ATTD of dietary CP, DM, OM, NDF in lactating sows decreased linearly and quadratically when the substitution level of FFRB increased gradually. Consistent with results in gestating sows, high dietary fiber depressed the digestibility of conventional nutrients in lactating sows (Noblet et al. 2001). However, an appropriate increase in dietary fiber can enhance the ATTD (Kass et al. 1980; Li et al. 2018). In addition, lactating sows also possess a certain tolerance to dietary fiber. The ATTD of ADF of FFRB in lactating sows exhibited a significant quadratic response to the increasing substitution level, with the maximum digestibility attained at the 25% substitution level. Based on the results of Exp. 1 and Exp. 3, beyond the 35% and 25% substitution levels for gestating and lactating sows, respectively, the increased fiber content may have exceeded their tolerance capacities.

The average ATTD of NDF in FFRB (50.9%) in lactating sows in Exp. 3 exceeded that reported in a previous study for gestating sows (36.68%) (Casas and Stein 2017), who showed that the level of feed intake did not affect the ATTD of nutrients or the concentration of available energy of a corn-soybean meal diet or diets containing FFRB or defatted rice bran. Therefore, it is generally believed that feeding amount does not affect the evaluation of the nutritional value of feedstuffs in sows. By excluding the effect of feed intake, it can be more conclusively determined that distinct physiological stages are the main reason for the differences in nutrient ATTD. Compared with gestating sows, lactating sows show higher nutrient digestibility and utilization due to increased lactation requirements (Casas and Stein 2017; Liu et al. 2021). The higher ATTD of most nutrients for diets and FFRB observed in lactating sows (Exp. 3), compared with gestating sows in Exp. 1 further validates the aforementioned conclusions.

### Effects of substitution levels on energy balance and available energy in diets and FFRB for gestating and lactating sows

Notably, feed intake exerts no interference on intestinal nutrient digestion and feed energy value determination, yet it substantially regulates whole-body energy retention and metabolic status of sows, which can be clearly reflected in the energy balance data of gestating sows in Exp. 1. Sows were fed at 1.5 times the maintenance requirement, but RE_L_ in all groups was negative, which may be due to the low feeding level failing to meet the normal nutritional needs of sows. When nutritional intake is insufficient to meet actual requirements, sows will oxidize body fat to supplement energy (Chwalibog et al. 2004). Meanwhile, the RQ for sows in the fed state was ≤ 1, indicating that nutrient intake was insufficient to meet maintenance requirements and that gestating sows were in a catabolic state with fat likely serving as the primary oxidative substrate. Therefore, it may be necessary to increase energy intake levels to regulate this imbalance and ensure the nutritional needs of the pregnant sows are met in future study.

Higher nutrient ATTD in the 35% substitution group indicated that gestating sows achieved greater digestion and utilization of FFRB, thereby resulting in the highest RE_L_, RE, and NE/ME. In addition, the CV of available energy values of FFRB in the 35% substitution level group was the lowest. It is generally accepted that a lower CV indicates greater stability of the measured available energy values, with improved data repeatability and higher accuracy (Dong 2020). Based on the ATTD of nutrients, available energy, and CVs of FFRB, it is recommended that 35% FFRB be used to replace the energy-supplying components in the basal diet for gestating sows.

Consistent with gestating sows, the CV of available energy values was also a key indicator for determining the optimal FFRB substitution level for lactating sows. The CV of DE and ME of FFRB in the 15% substitution group was 6.26% and 9.55%, and 5.41% and 9.18% higher than that in the 25% and 35% substitution groups, respectively. A previous study had shown that when using the substitution method to formulate diets for determining the available energy values of ingredients, an excessively low substitution level may increase the impact of other energy-supplying components in the diet on the test ingredient, thereby increasing the CV of available energy values of the test ingredient (Dong 2020). In contrast, the 25% substitution group had the smallest CV of DE and ME values for FFRB and the highest energy utilization (ME/DE). Higher ATTD of nutrients and energy utilization, coupled with lower CV for available energy, indicated that 25% was the optimal substitution level for accurately determining available energy values of FFRB in lactating sows.

### Nutrient composition and ATTD of different FFRB samples for gestating and lactating sows

According to GB 10371-89, No.4 and No.11 FFRB were classified as first-grade rice bran (CP ≥ 13.0%, Ash < 8.0%), No.1, 3, 5, and 6 FFRB were classified as second-grade rice bran (CP ≥ 12.0%, Ash < 9.0%), Nos.2, 8, 9, and 10 FFRB were classified as third-grade rice bran (CP ≥ 11.0%; Ash < 10.0%) (General Administration of Quality Supervision and Inspection and Quarantine of the People’s Republic of China 1989). The CP content of all FFRB test samples ranged from 13.41% to 17.71%, and EE content ranged from 7.17% to 18.47%, indicating significant variations among different samples. Thus, it is inaccurate to use a static average value to represent the available energy values of all FFRB samples, and the establishment of a dynamic prediction model for the available energy values of FFRB is necessary.

The ATTD of ADF for FFRB No. 4 in gestating sows was significantly higher than that of samples Nos. 5 and No. 6. These significant differences in ATTD of ADF may be mainly attributed to different processing technologies of FFRB, which result in variations in fiber composition and cell wall structure, thereby affecting fiber degradation and utilization in the gastrointestinal tract of pigs (Huang et al. 2021). The ATTD of CP for FFRB No.6 in gestating sows was the lowest among all samples. The significant difference in ATTD of CP for FFRB in gestating sows might be related to the fiber contents of different FFRB samples. A previous study indicated that increasing dietary fiber levels reduced CP digestibility in pigs (Lyu et al. 2018), which was consistent with the results of Exp. 1. Except for No. 2, sample No. 6 exhibited the highest fiber content. However, the elevated EE content of the sample No. 2 partially mitigated the negative impact of high fiber on digestibility. In addition, different types of fiber have different effects on ATTD of CP, the content of soluble dietary fiber has no significant effect on ATTD of CP, whereas the content of insoluble dietary fiber significantly reduces ATTD of CP (Owusu-Asiedu et al. 2006; Renteria-Flores et al. 2008), which may collectively account for the lower ATTD of CP for No. 6 FFRB. No significant differences were found in ATTD of different FFRB samples for lactating sows, but the average ATTD of NDF, ADF, and CP of FFRB in lactating sows was numerically higher than that in gestating sows. Agreed with our results, lactating sows had greater ATTD of GE and NDF than gestating sows, regardless of whether diets were supplemented with FFRB or defatted rice bran, which may be attributed to the greater demand for milk production, resulting in upregulation of digestive enzymes or transporters in the small intestine and thereby enhancing nutrients absorption (Casas et al. 2022).

### Available energy values of different FFRB samples for gestating and lactating sows

The average DE, ME, and NE of five FFRB samples in gestating sows were 15.50, 14.89, 11.66 MJ/kg DM, respectively. These values were lower than the DE (17.02 MJ/kg DM), ME (16.55 MJ/kg DM), and NE (12.89 MJ/kg DM) values of FFRB (EE < 15%) for sows specified in Nutrient requirements for pigs in China (2020), but higher than DE (14.16 MJ/kg DM), ME (13.69 MJ/kg DM), and NE (10.42 MJ/kg DM) values of FFRB for pigs reported in NRC (2012). This might be related to the different growth stages of swine and different types of FFRB samples (Kass et al. 1980; Lyu et al. 2018).

The average DE value (13.72 MJ/kg DM) for FFRB samples in lactating sows was slightly lower than that (14.16 MJ/kg DM) reported by NRC (2012) for swine. In addition to the differences caused by the different physiological stages, FFRB from different sources or processed by different methods may also exhibit variations in nutritional value. The ME range for FFRB was 11.13–12.19 MJ/kg DM in lactating sows, slightly higher than that (11.09 MJ/kg DM) determined in growing pigs (Nehring and Haenlein 1973); however, it was lower than the ME value (13.69 MJ/kg DM) tabulated in NRC (2012) for swine. This discrepancy may be attribute to the differences in nutrient composition of FFRB samples. Therefore, accurately determining the available energy values of various ingredients for different physiological states can facilitate precise diets formulation that meets the nutritional requirements of sows (Brooks and Lumanta 1975; Campabadal et al. 1976; Liu et al. 2021). A previous study reported that the lower the fiber content of ingredients, the higher their available energy values (Lyu 2021). FFRB No.8 had the lowest NDF and ADF content, as well as the highest measured DE and ME values of FFRB for lactating sows, which was consistent with previous reports (Lyu 2021). A comparison of ME/DE of FFRB between gestating and lactating sows revealed that FFRB had higher energy utilization in gestating sows. The faster digesta passage rate associated with ad libitum feeding reduces the efficiency of energy utilization in lactating sows. The higher heat increment and intense energy demand for milk synthesis decrease the net energy retention in lactating sows (Noblet et al. 1990). The greater energy retention efficiency in gestating sows under restricted feeding results in energy being primarily deposited as body fat with minimal energy losses (Whittemore 1998).

Therefore, to achieve accurate utilization of ingredients in sows, it is necessary to evaluate ingredients separately for gestating and lactating physiological stages. In addition, given the variations in nutritional value among different ingredient types, their efficient utilization cannot be achieved solely through fixed available energy values; thus, dynamic prediction equations for available energy should be established.

### Prediction equations of available energy values of different FFRB samples for gestating and lactating sows

Using conventional chemical compositions to predict the available energy of ingredients serves as an approach to establish prediction equations for available energy (Noblet et al. 1993). NDF and ADF were included as predictor variables in available energy prediction equations for gestating sows, which was consistent with the conclusion that fiber affects the energy value and digestibility of ingredients (Noblet and van Milgen 2004; Chen et al. 2023). The available energy prediction equation with the maximum *R*^2^, the minimum RMSE and *P *< 0.05 was selected as the optimal prediction equation. The DE value of FFRB for gestating sows showed a significant positive correlation with EE content. Previous reports indicated that EE content of ingredient affects available energy values (Bruce et al. 2006; Mogielnicka-Brzozowska et al. 2010; Maison et al. 2015). Therefore, EE was included as the main predictor variable in the DE regression equation. Ash was incorporated into the ME prediction equation for FFRB, which was similar to the equation established by Noblet et al. (1994). Starch is the main component of carbohydrates in the diet and the primary precursor for pigs to obtain glucose for metabolism. The ADF content of FFRB had a strong correlation with its NE value; thus, ADF and ST contents of FFRB were used as predictor variables in the NE prediction equation for FFRB. Although ADF content of FFRB had a strong correlation with ME, it was not included in the ME prediction equation. This indicates that to establish more comprehensive and representative prediction equations for the available energy of FFRB for gestating sows, it is necessary to increase the number of FFRB samples and improve the selection of predictor variables by accumulating more experimental data.

A previous study showed a negative correlation between NDF, ADF, and other indicators in cereal by-products (Chen et al. 2016), which was consistent with the results of this article. Ash of FFRB was significantly negatively correlated with GE, whereas EE of FFRB was significantly positively correlated with GE. These finding were consistent with the results of previous study on the correlation between GE and Ash and EE (Lyu et al. 2023). Based on *R*^2^ and *P* value, the ME prediction equation for FFRB was established using NDF, CP, and DE as predictors in Exp. 4.

The chemical composition of the five FFRB samples in Exp. 2 was input into the prediction equations for gestating sows. The predicted DE values (15.59, 15.15, 15.75, 15.20, and 15.05 MJ/kg DM), predicted ME values (14.97, 14.68, 15.12, 14.71, and 14.61 MJ/kg DM) and predicted NE values (10.77, 12.11, 12.84, 11.85, and 11.62 MJ/kg DM) were close to the corresponding tabulated values of sows in Table 13, indicating that the established prediction equations of available energy of FFRB for gestating sows performed well in terms of prediction. The chemical composition of rice bran for swine listed in NRC (2012) was applied to the established prediction equations for the available energy of FFRB for gestating sows. The predicted values (DE, 14.93 MJ/kg DM; ME, 14.35 MJ/kg DM; NE, 11.43 MJ/kg DM) were all higher than recommend values in NRC (2012) (DE, 14.16 MJ/kg DM; ME, 13.69 MJ/kg DM; NE, 10.42 MJ/kg DM) for swine. This discrepancy may be attributed to the greater EE content of FFRB used in the present study compared with that in NRC (2012), thereby resulting in higher available energy values, which is consistent with the finding by Casas et al. (2022) that EE content is a key factor affecting the available energy of rice bran in sows. In addition, distinct physiological states of gestating sows in this article versus swine in NRC (2012) may result in variations in the available energy value of FFRB. The chemical composition of high-oil FFRB from Nutrient Requirements of Swine in China (2020) was input into the prediction equations for gestating sows. The predicted values (DE, 17.99 MJ/kg DM; ME, 16.67 MJ/kg DM; NE, 12.80 MJ/kg DM) were close to the corresponding tabulated values for sows (DE, 17.10 MJ/kg DM; ME, 16.66 MJ/kg DM; NE, 12.98 MJ/kg DM), indicating that the established prediction equations exhibited favorable predictive performance.

By substituting the nutrient composition of the four FFRB samples in Exp. 4 into the ME prediction equation for FFRB of lactating sows, the calculated values (12.19, 11.50, 11.13, and 11.71 MJ/kg DM) were found to be close to the measured values presented in Table 14, except for No. 10 FFRB. The chemical compositions of rice bran from NRC (2012) and Nutrient Requirements of Swine in China (2020) were introduced into the available energy prediction equations for lactating sows, the predicted values were lower than the reported values in both standards for swine and sows. Although lactating sows had greater nutrient digestibility of FFRB (Casas et al. 2022), intensive energy expenditure for milk synthesis, along with the shorter retention time of digesta in the gastrointestinal tract due to ad libitum feeding (Noblet et al. 1990), resulted in lower available energy values, which aligns with the energy utilization characteristics of lactating sows reported by Whittemore (1998). The prediction equation for DE of FFRB in lactating sows was not presented in this manuscript due to its relatively low *R*^2^. Therefore, the range of FFRB sources must be expanded so that more representative predictive equations for available energy of FFRB in pregnant and lactating sows can be derived and subsequently validated through animal experiments.

## Conclusions

Ingredient types and physiological stages affect available energy values of ingredients. Excessive dietary fiber content reduces the ATTD of nutrients, whereas dietary EE supplementation can partially counteract the adverse effects of fiber on ATTD. Lactating sows demonstrated superior digestive capacity compared with gestating sows, however, the energy utilization of FFRB was more efficient in gestating sows. Based on the CV of available energy, the ATTD of nutrients and available energy values, the recommended substitution level of FFRB for energy-supplying components in the basal diet was 35% for gestating sows and 25% for lactating sows, using the substitution method. Fiber components, EE, and CP are the primary predictors of available energy values for FFRB in sows. By establishing dynamic prediction equations for FFRB, precise diet formulation and improved feed utilization can be achieved.

## Supplementary Material

skag267_Supplementary_Data

## Abbreviations

ADF: acid detergent fiber
ANOVA: analysis of variance
Ash: crude ash
ATTD: apparent total tract digestibility
CH_4_E: CH_4_ energy
CP: crude protein
DE: digestible energy
DM: dry matter
DMI: dry matter intake
EE: ether extract
FFRB: full-fat rice bran
FHP: fasting heat production
GE: gross energy
ME: metabolizable energy
NDF: neutral detergent fiber
NE: net energy
OM: organic matter
R: multiple coefficient of determination
RE: energy retention
RE_L_: energy retention as lipid
RE_P_: energy retention as protein
RMSE: root mean square error
RQ: respiratory quotient
ST: starch
THP: total heat production
UE: urine energy
