# Supplementary material for: Available energy and nutrient apparent total tract digestibility of full-fat rice bran fed to gestating and lactating sows
Source: J Anim Sci. 2026 Aug 26;104:skag267. doi: 10.1093/jas/skag267 (PMC13600676; doi:10.1093/jas/skag267)
Supplement: Supplementary file 1 [file skag267_supplementary_data.docx]

Table S1. ATTD of nutrients and nitrogen balance of different diets fed to gestating sows in Exp. 2

| Items^1^ | FFRB diets^2^ | | | | | | SEM | *P* value |
| --- | --- | --- | --- | --- | --- | --- | --- | --- |
|  | Basal diet | D2 | D3 | D4 | D5 | D6 |  |  |
| DMI (kg/d) | 2.43 | 2.41 | 2.4 | 2.42 | 2.47 | 2.47 | 0.02 | 0.27 |
| ATTD (%) | | | | | | | | |
| DM | 92.1^a^ | 83.1^b^ | 84.8^b^ | 85.4^b^ | 83.0^b^ | 83.9^b^ | 0.71 | < 0.01 |
| CP | 89.7^a^ | 82.7^b^ | 84.2^b^ | 84.7^b^ | 83.5^b^ | 84.3^b^ | 0.64 | < 0.01 |
| NDF | 65.2^a^ | 43.8^bc^ | 38.4^bc^ | 29.6^c^ | 35.2^bc^ | 51.5^ab^ | 2.82 | < 0.01 |
| ADF | 72.9^a^ | 45.4^bc^ | 41.0^bc^ | 48.9^b^ | 33.5^c^ | 42.7^bc^ | 2.62 | < 0.01 |
| EE | 64.3^b^ | 79.5^a^ | 77.7^a^ | 76.9^a^ | 75.3^a^ | 76.8^a^ | 1.14 | < 0.01 |
| OM | 93.8^a^ | 86.3^b^ | 87.7^b^ | 88.4^b^ | 86.3^b^ | 86.9^b^ | 0.53 | < 0.01 |
| Nitrogen balance (g/d) | | | | | | | | |
| Intake | 52.85^c^ | 53.56^c^ | 54.38^c^ | 56.01^bc^ | 57.49^b^ | 59.98^a^ | 0.79 | < 0.01 |
| Feces output | 5.46^b^ | 9.27^a^ | 8.60^a^ | 8.56^a^ | 9.47^a^ | 9.42^a^ | 0.42 | < 0.01 |
| Urine output | 22.38 | 18.69 | 18.59 | 25.24 | 23.09 | 17.22 | 0.86 | 0.31 |
| Retention | 25.01 | 25.60 | 27.19 | 22.21 | 24.93 | 33.34 | 1.28 | 0.15 |

ATTD, apparent total tract digestibility; FFRB, full-fat rice bran. SEM, standard error of the mean.

^1^DMI, dry matter intake; DM, dry matter; CP, crude protein; NDF, neutral detergent fiber; ADF, acid detergent fiber; EE, ether extract; OM, organic matter.

^2^D2, D3, D4, D5 and D6 were test diets containing 35% FFRB from 5 different sources.

Different lowercase letters within the same row indicate significant differences (*P* < 0.05), while the same or no letters indicate no significant differences (*P* > 0.05).

Table S2. Energy balance of different diets fed to gestating sows in Exp. 2

| Items^1^ | Basal diet | FFRB diets^2^ | | | | | SEM | *P* value |
| --- | --- | --- | --- | --- | --- | --- | --- | --- |
|  |  | D2 | D3 | D4 | D5 | D6 |  |  |
| Energy balance (kJ/kg BW^0.75^/d) | | | | | | | | |
| ME intake | 690.50^a^ | 607.70^b^ | 627.55^b^ | 626.17^b^ | 596.11^b^ | 621.62^b^ | 6.86 | < 0.01 |
| THP | 501.52 | 488.74 | 479.58 | 447.92 | 449.14 | 490.92 | 9.93 | 0.53 |
| FHP | 365.83 | 374.47 | 347.24 | 328.43 | 336.87 | 378.45 | 8.33 | 0.42 |
| RE_P_ | 69.62 | 70.17 | 74.6 | 59.58 | 65.37 | 86.92 | 3.19 | 0.22 |
| RE_L_ | 119.36 | 48.79 | 73.37 | 118.67 | 81.60 | 43.79 | 10.47 | 0.14 |
| RE | 188.98 | 118.96 | 147.97 | 178.25 | 146.97 | 130.71 | 10.44 | 0.36 |
| RQ |  |  |  |  |  |  |  |  |
| Fed state | 1.04 | 1.02 | 1.03 | 1.03 | 1.05 | 1.01 | 0.01 | 0.79 |
| Fasted state | 0.85 | 0.8 | 0.82 | 0.88 | 0.85 | 0.82 | 0.01 | 0.61 |
| Available energy values (MJ/kg DM) | | | | | | | | |
| DE | 16.23 | 16.00 | 15.87 | 16.06 | 15.92 | 15.78 | 0.07 | 0.18 |
| ME | 14.62 | 14.35 | 14.12 | 14.45 | 14.38 | 13.99 | 0.13 | 0.21 |
| NE | 11.11 | 10.21 | 10.12 | 10.38 | 10.44 | 10.02 | 0.14 | 0.2 |
| Energy utilization (%) | | | | | | | | |
| UE/DE | 9.03 | 9.44 | 10.27 | 9.36 | 8.84 | 10.36 | 0.32 | 0.75 |
| CH_4_E/DE | 0.89 | 0.87 | 0.76 | 0.66 | 0.83 | 0.98 | 0.21 | 0.14 |
| ME/DE | 90.08 | 89.69 | 88.97 | 89.98 | 90.33 | 88.66 | 0.58 | 0.26 |
| NE/ME | 75.99 | 71.15 | 71.67 | 71.83 | 72.60 | 71.62 | 0.73 | 0.31 |

FFRB, full-fat rice bran; SEM, standard error of the mean.

^1^THP, total heat production; FHP, fasting heat production; RE_P_, energy retention as protein; RE_L_, energy retention as lipid; RE, energy retention; RQ, respiratory quotient; DE, digestible energy; ME, metabolizable energy; NE, net energy; UE, urine energy; CH_4_E, CH_4_ energy.

^2^D2, D3, D4, D5 and D6 were test diets containing 35% FFRB from 5 different sources.

Different lowercase letters within the same row indicate significant differences (*P* < 0.05), while the same or no letters indicate no significant differences (*P* > 0.05).

Table S3. Effects of various treatments on nutrient ATTD and available energy value of diets fed to lactating sows in Exp. 4 (%, DM basis)

| Items^1^ | Basal diet | FFRB diets | | | | SEM | *P* value |
| --- | --- | --- | --- | --- | --- | --- | --- |
|  |  | No.8 | No.9 | No.10 | No.11 |  |  |
| DMI (kg/d) | 7.24 | 7.04 | 7.4 | 7.33 | 7.1 | 0.21 | 0.75 |
| ATTD (%) | | | | | | | |
| CP | 91.6^a^ | 87.5^b^ | 86.1^b^ | 86.9^b^ | 83.7^b^ | 0.73 | < 0.05 |
| DM | 91.9^a^ | 84.0^b^ | 82.5^b^ | 82.7^b^ | 84.7^b^ | 0.91 | < 0.05 |
| OM | 93.4^a^ | 87.2^b^ | 85.2^b^ | 86.3^b^ | 87.6^b^ | 0.67 | < 0.05 |
| NDF | 73.1^a^ | 56.3^b^ | 53.3^b^ | 58.9^b^ | 61.0^b^ | 2.14 | < 0.05 |
| ADF | 78.1^a^ | 53.6^c^ | 50.5^d^ | 57.9^b^ | 53.7^c^ | 3.83 | < 0.05 |
| Available energy values (MJ/kg DM) | | | | | | | |
| DE | 14.52^a^ | 13.72^c^ | 13.39^c^ | 13.86^bc^ | 14.37^ab^ | 0.12 | < 0.05 |
| ME | 13.97^a^ | 13.18^ab^ | 12.84^b^ | 13.38^ab^ | 13.91^a^ | 0.13 | < 0.05 |
| Energy utilization (%) | | | | | | | |
| ME/DE | 96.21 | 96.06 | 95.89 | 96.54 | 96.8 | 0.01 | 0.8 |

ATTD, apparent total tract digestibility; FFRB, full-fat rice bran; SEM, standard error of the mean.

^1^DMI, dry matter intake; CP, crude protein; DM, dry matter; OM, organic matter; NDF, neutral detergent fiber; ADF, acid detergent fiber; DE, digestible energy; ME; metabolizable energy.

Different lowercase letters within the same row indicate significant differences (*P* < 0.05), while the same or no letters indicate no significant differences (*P* > 0.05).

Table S4. Correlation analysis of routine chemical composition and available energy values of five FFRB samples fed to gestating sows in Exp. 2

| Items^1^ | NE | DE | ME | GE | CP | ST | EE | NDF | ADF | Ash |
| --- | --- | --- | --- | --- | --- | --- | --- | --- | --- | --- |
| NE | 1 |  |  |  |  |  |  |  |  |  |
| DE | 0.18 | 1 |  |  |  |  |  |  |  |  |
| ME | 0.57 | 0.85 | 1 |  |  |  |  |  |  |  |
| GE | 0.32 | 0.56 | 0.2 | 1 |  |  |  |  |  |  |
| CP | 0.03 | -0.61 | -0.44 | -0.91* | 1 |  |  |  |  |  |
| ST | 0.21 | 0.15 | 0.33 | -0.43 | 0.04 | 1 |  |  |  |  |
| EE | 0.29 | 0.94* | 0.44 | 0.69 | -0.5 | -0.63 | 1 |  |  |  |
| NDF | -0.82 | 0.06 | -0.55 | 0.33 | 0.08 | -0.92* | 0.49 | 1 |  |  |
| ADF | -0.92* | -0.34 | -0.76 | 0.37 | -0.01 | -0.81 | 0.19 | 0.87 | 1 |  |
| Ash | -0.49 | -0.33 | -0.66 | 0.45 | -0.18 | -0.69 | 0.07 | 0.64 | 0.93* | 1 |

FFRB, full-fat rice bran.

^1^NE, net energy; DE, digestible energy; ME; metabolizable energy; GE, gross energy;

CP, crude protein; ST, starch; EE, ether extract; NDF, neutral detergent fiber; ADF, acid detergent fiber; Ash, crude ash.

**P* < 0.05.

Table S5. Correlation analysis of routine chemical compositions and available energy values of four FFRB samples fed to lactating sows in Exp. 4

| Items^1^ | DE | ME | GE | CP | EE | NDF | ADF | Ash |
| --- | --- | --- | --- | --- | --- | --- | --- | --- |
| DE | 1 |  |  |  |  |  |  |  |
| ME | 0.99* |  |  |  |  |  |  |  |
| GE | 0.16 | 0.04 | 1 |  |  |  |  |  |
| CP | 0.32 | 0.19 | 0.68 | 1 |  |  |  |  |
| EE | 0.3 | 0.19 | 0.98* | -0.64 | 1 |  |  |  |
| NDF | -0.19 | -0.09 | -0.61 | -0.36 | -0.72 | 1 |  |  |
| ADF | -0.27 | -0.18 | -0.62 | -0.35 | -0.74 | 0.99* | 1 |  |
| Ash | 0.09 | 0.18 | -0.91* | 0.5 | -0.81 | 0.27 | 0.27 | 1 |

FFRB, full-fat rice bran.

^1^DE, digestible energy; ME, metabolizable energy; GE, gross energy; CP, crude protein; EE, ether extract; NDF, neutral detergent fiber; ADF, acid detergent fiber; Ash, crude ash.

**P* < 0.05.

Table S6. The content of nutrients of FFRB in NRC (2012) and Nutrient Requirements of Swine in China (2020) (%, DM basis)

| Items^1^ | NRC (2012) | Nutrient Requirements of Swine in China (2020) |
| --- | --- | --- |
| EE | 15.03 | 19.66 |
| NDF | 28.69 | 26.31 |
| ADF | 12.96 | 9.17 |
| Ash | 16.16 | 8.21 |
| ST | 29.48 | 28.94 |

FFRB, full-fat rice bran.

^1^EE, ether extract; NDF, neutral detergent fiber; ADF, acid detergent fiber; Ash, crude ash; ST, starch.

Table S7. The prediction content of available energy for FFRB in NRC (2012) and Nutrient Requirements of Swine in China (2020) (%, DM basis)

| Items^1^ | NRC (2012) | Nutrient Requirements of Swine in China (2020) |
| --- | --- | --- |
| DE | 14.93 | 17.99 |
| ME | 14.35 | 16.67 |
| NE | 11.43 | 12.80 |

FFRB, full-fat rice bran.

^1^DE, digestible energy; ME, metabolizable energy; NE, net energy.
